# Supplementary material for: Causal Relationship between Aging and Anorexia Nervosa: A White-Matter-Microstructure-Mediated Mendelian Randomization Analysis
Source: Biomedicines. 2024 Aug 16;12(8):1874. doi: 10.3390/biomedicines12081874 (PMC11351342; doi:10.3390/biomedicines12081874)
Supplement: Supplementary file 1 [file biomedicines-12-01874-s001.zip › Supplementary Figure/Figure S4/Singe SNP plot of MR analysis for TL to ubm-b-1648.pdf]

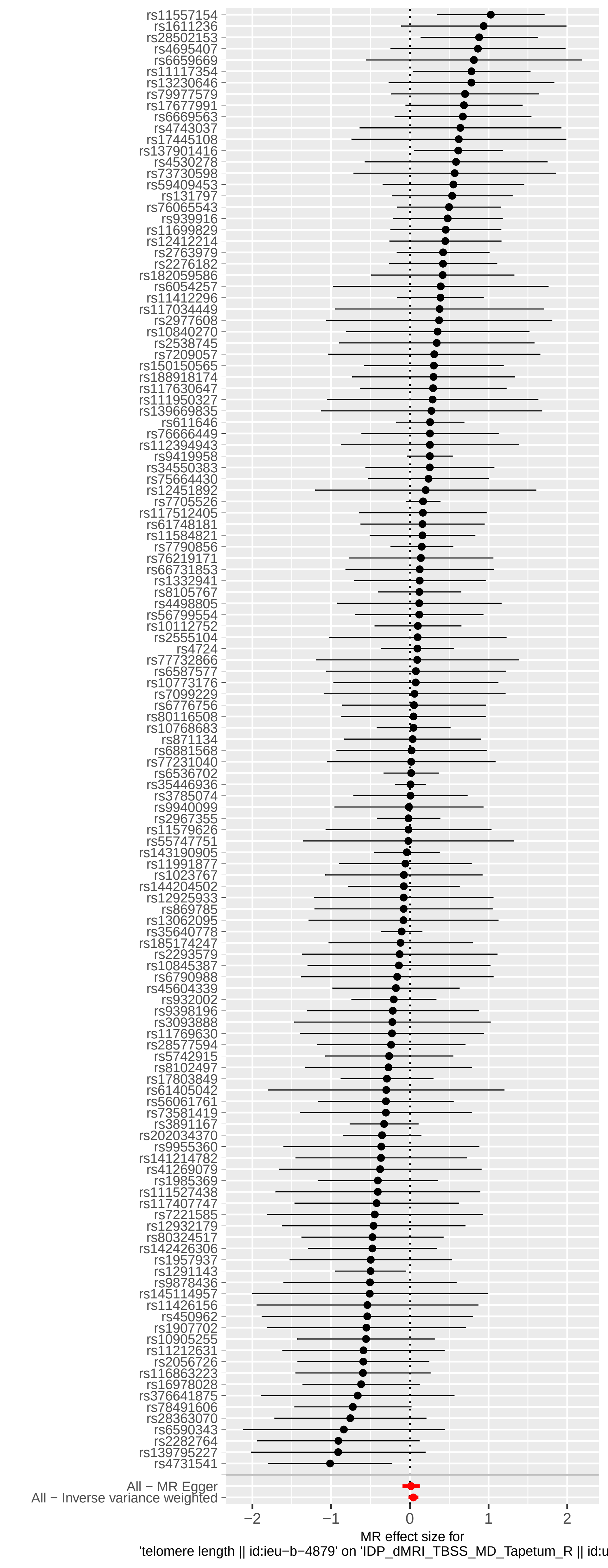
rs11557154
rs1611236
rs28502153
rs4695407
rs6659669
rs11117354
rs13230646
rs79977579
rs17677991
rs6669563
rs4743037
rs17445108
rs137901416
rs4530278
rs73730598
rs59409453
rs131797
rs76065543
rs939916
rs11699829
rs12412214
rs2763979
rs2276182
rs182059586
rs6054257
rs11412296
rs117034449
rs2977608
rs10840270
rs2538745
rs7209057
rs150150565
rs188918174
rs117630647
rs111950327
rs139669835
rs611646
rs76666449
rs112394943
rs9419958
rs34550383
rs75664430
rs12451892
rs7705526
rs117512405
rs61748181
rs11584821
rs7790856
rs76219171
rs66731853
rs1332941
rs8105767
rs4498805
rs56799554
rs10112752
rs2555104
rs4724
rs77732866
rs6587577
rs10773176
rs7099229
rs6776756
rs80116508
rs10768683
rs871134
rs6881568
rs77231040
rs6536702
rs35446936
rs3785074
rs9940099
rs2967355
rs11579626
rs55747751
rs143190905
rs11991877
rs1023767
rs144204502
rs12925933
rs869785
rs13062095
rs35640778
rs185174247
rs2293579
rs10845387
rs6790988
rs45604339
rs932002
rs9398196
rs3093888
rs11769630
rs28577594
rs5742915
rs8102497
rs17803849
rs61405042
rs56061761
rs73581419
rs3891167
rs202034370
rs9955360
rs141214782
rs41269079
rs1985369
rs111527438
rs117407747
rs7221585
rs12932179
rs80324517
rs142426306
rs1957937
rs1291143
rs9878436
rs145114957
rs11426156
rs450962
rs1907702
rs10905255
rs11212631
rs2056726
rs116863223
rs16978028
rs376641875
rs78491606
rs28363070
rs6590343
rs2282764
rs139795227
rs4731541
All − MR Egger
All − Inverse variance weighted
−2
−1
0
1
2
MR effect size for
'telomere length || id:ieu-b-4879' on 'IDP_dMRI_TBSS_MD_Tapetum_R || id:u
